# Do preterm-born adolescents have a poorer oral health-related quality of life?

**DOI:** 10.1186/s12903-021-01799-3

**Published:** 2021-09-09

**Authors:** Susanne Brogårdh-Roth, Liselotte Paulsson, Pernilla Larsson, Ewacarin Ekberg

**Affiliations:** 1 Department of Pediatric Dentistry, Faculty of Odontology, Malmö University, 205 06, Malmö, Sweden; 2 Department of Orthodontics, Faculty of Odontology, Malmö University, Malmö, Sweden; 3 Department of Prosthodontics, Faculty of Odontology, Malmö University, Malmö, Sweden; 4 Centre of Oral Rehabilitation, Folktandvården Östergötland, Linköping, Sweden; 5 Department of Orofacial Pain and Jaw Function, Faculty of Odontology, Malmö University, Malmö, Sweden

**Keywords:** Oral health-related quality of life, Adolescents, Born preterm

## Abstract

**Background:**

To evaluate oral health-related quality of life (OHRQoL) over a period of five years using the Oral Health Impact Profile (OHIP-14) questionnaire in a population of Swedish adolescents born preterm and full term.

**Methods:**

In a longitudinal study of adolescents aged 12–14 and 17–19, changes over time in OHRQoL were measured by using OHIP-14. The OHIP-14 score, self-reported chronic illness, temporomandibular disorder (TMD pain) and subjective orthodontic treatment need were compared between 98 extremely and very preterm born (< 32 gestational week) and 93 full-term controls (≥ 37 gestational week) at two ages. The chi-square test was used for comparisons within the extremely-, very-, and full-term control groups, and to contrast the differences of mean scores of OHIP-14, the ANOVA test was used for comparisons within the study groups of extremely preterm, very preterm and full term-born adolescents.

**Results:**

All adolescents reported a good self-perceived OHRQoL. No significant differences in the comparisons of the total mean scores were revealed between the groups, between gender or in domain-specific scores over the 5-year period. Very preterm adolescents with reported chronic illness at 12–14 years of age showed significantly higher mean scores of OHIP-14 compared with those without chronic illness (*p* = *0.015*). At age 17–19, significantly higher mean scores of OHIP-14 were reported by very preterm adolescents with TMD pain compared to those without TMD pain (*p* = *0.024*). Significantly higher mean scores of OHIP-14 were found among the extremely preterm (*p* = *0.011*) and very preterm born adolescents (*p* = *0.031*) with a subjective need of orthodontic treatment compared with those without orthodontic treatment need.

**Conclusions:**

Poor OHRQoL measured with OHIP-14 in very preterm adolescents aged 12–14 was related to chronic illness and aged 17–19 to TMD pain. In addition, extremely and very preterm-born adolescents with subjective orthodontic treatment need at 17–19 years of age also reported poor OHRQoL. To improve the dentist–patient relationship and achieve more successful treatment results, it is important for dental clinicians to understand the impact that chronic illness, TMD pain and orthodontic treatment need has on OHRQoL in preterm-born adolescents.

## Background

In recent years, researchers have been increasingly interested in quality of life as a way to understand individual well-being and self-esteem and as a way to identify factors that impact this multidimensional perspective of life satisfaction. Given that preterm-born individuals often have a compromised health status with lifelong consequences for health, growth, and development, concerns about the long-term well-being of the preterm child and family have been expressed [1, 2]. A recent review suggested that prematurity itself may be considered a chronic condition, with problems that can persist into late adolescence and adulthood [1]. Preterm-born individuals have been found to have a greater risk for neuro-psychological and behavioural problems and also cardiovascular disease, including elevated blood pressure and metabolic syndromes [1, 3]. The personal burden of different diseases and impairments in addition to certain socio-economic disadvantages may contribute to a reduced life quality in children and young people [4, 5]. Therefore, it is important to follow the individual’s progress through adolescence into adulthood. Today, several studies have examined health-related quality of life (HRQoL) to evaluate long-term physical, emotional and social functioning after preterm birth, with contradictory results. A systematic review found various degrees of lower health-related quality of life in preterm and very low birth weight (VLBW) children, starting from preschool age and into young adulthood, compared with full-term controls [6]. However, a Swedish study reported that preterm adolescents at 18 years of age did not differ from full-term controls regarding quality of life or expectations for the future [7]. Another study of preterm-born young adults also reported no change in quality of life of those between 19 and 28 years [8]. However, a recent study has revealed that in the extremely preterm-born group, quality of life, and in particular, psychological health, deteriorated from adolescence to young adulthood [9]. Furthermore, a lower quality of life in very preterm individuals has been associated with economic- and social-functioning problems in adulthood [5].

Orofacial function and health is another area to address when measuring satisfaction with life. One’s quality of life in relation to oral and general health can be measured by the notion of Oral Health-Related Quality of Life (OHRQoL). The idea to use the patient’s own perception of their oral health status stems from the need to assess the outcome of clinical interventions and predict treatment needs, which is essential for the allocation of health resources. Children and adolescents born preterm constitute a new group of patients for which the dental team needs to increase and deepen its knowledge. Various methods can be used to analyse OHRQoL, and that which is most extensively used is a multiple-item questionnaire—the Oral Health Impact Profile (OHIP-14)—which is a valid instrument used globally to measure the concept of OHRQoL [10]. This instrument has been translated into Swedish [11] and has been validated by Larsson et al. [12], which gives the translation excellent reliability and validity. In the Dimensions of Oral Health-Related Quality of Life (DOQ) Project, an international study of general population, the dimensions of OHRQoL has been evidence-based determined and a new four-dimensional structure established. OHRQoL consists of four highly correlated factors—Oral Function, Oro-facial Pain, Oro-facial Appearance and Psychosocial Impact [13, 14]. To our knowledge, no studies are available on health-related quality of life (OHRQoL) measured with OHIP-14 in preterm-born individuals. The perspective of OHRQoL highlights important aspects of various oral conditions of interest and mainly concerns young people. One aspect that was highlighted was problems with eating due to tooth pain or temporomandibular disorders (TMD). In line with this, a systematic review found a substantial impact on quality of life in TMD patients [15]. Only one study in their review used OHIP-14 for those aged 16 years and above, and it showed significantly higher scores on OHIP-14 mean scores in TMD patients [16]. In addition, other studies have revealed that young people experience problems and discomfort with social interaction, and issues relating to emotional and psychological function due to malocclusions [17]. Extremely and very preterm children have been reported to have more malocclusion traits and a higher professional-assessed need for orthodontic treatment than full term-born children [18]. In a systematic review, it was found that, in general, malocclusions have a negative effect on children’s and adolescents’ OHRQoL, particularly when the malocclusions are present in the aesthetic zone [19].

Oral health is important for general health and well-being in all stages of life, and healthcare professionals need to take a holistic approach to the patient in routine health care, especially when the intention is to improve oral health, including managing the psychosocial aspects of health. The aim of this study was to describe OHRQoL over a 5-year perspective, starting at the age of 12–14 and then again at 17–19 years, by using the OHIP-14 questionnaire in a population of adolescents born preterm (extremely and very preterm) and full term. Another aim was to test how chronic illness, TMD pain and subjective orthodontic treatment need can influence the OHRQoL of preterm-born adolescents.

### Hypotheses

- Impaired OHRQoL at 12–14 years of age is predicted for impaired OHRQoL at 17–19 years of age in extremely and very preterm-born adolescents due to their higher rates of medical health problems (chronic illness, general health problems and daily medication).
- Adolescents with TMD pain have higher scores of OHRQoL than those adolescents without TMD pain, regardless of group.
- Adolescents with a subjective orthodontic treatment need have higher scores of OHRQoL than those without subjective orthodontic treatment need, regardless of group.

## Methods

### Study area

This study was conducted in the southwest part of the county of Skåne in southern Sweden. The preterm adolescents and the full-term controls included were born at the University Hospitals of Malmö and Lund in the same county (Skåne).

### Study design and participants

**Fig. 1:**
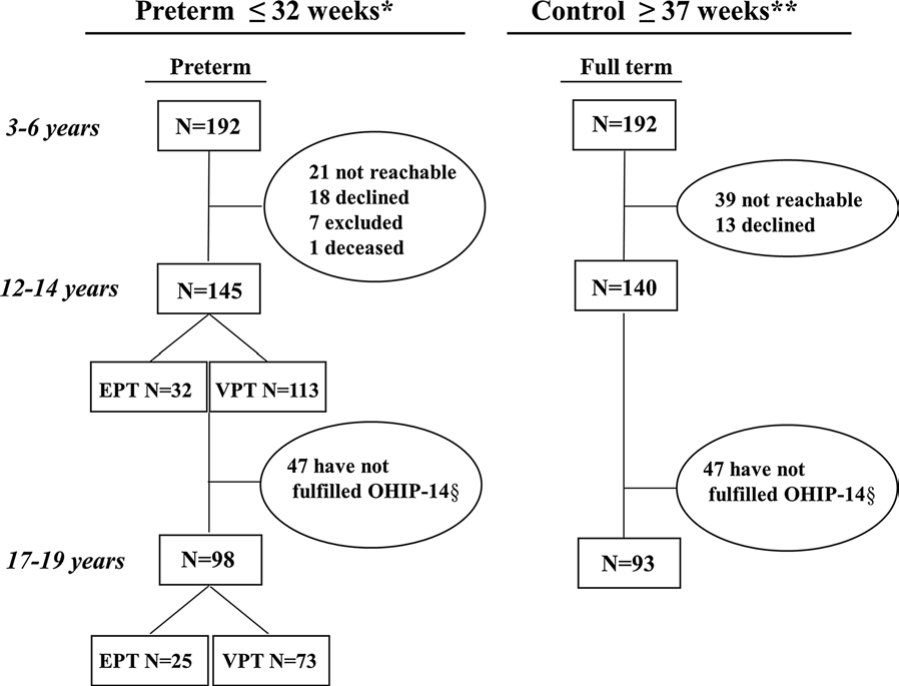
Flowchart including adolescents in the preterm group (extremely and very preterm-born) and the full-term control group from the start and then at the ages of 12–14 and 17–19. The adolescents who participated in the study had registered their oral-health related quality of life in OHIP-14 at both occasions as an adolescent. ^*^ Identified through the Swedish Medical Birth Register ^**^ Identified through dental clinics. Declined = Declined participation for reasons unknown. Excluded = Excluded owing to intellectual disability. Not fulfilled OHIP-14 § = The study design changed to a longitudinal design, and individuals who did not participate at both 12–14 year and 17–19 years of age were excluded. EPT = Extremely preterm (born in gestational weeks 23–28). VPT = Very preterm (born in gestational weeks 29–32)

Both the preterm and full-term participants originate from previous studies by Brogårdh-Roth et al. [20, 21]. They were again invited to participate in a questionnaire study at 12–14 and 17–19 years of age. The current study was of a longitudinal study design and included all the preterm and full-term adolescents who participated when they were both 12–14 and 17–19 years old. This group constituted the sample in the present study. Figure 1 shows the flowchart describing the study population of extremely preterm (EPT), very preterm (VPT), and full term-born adolescents that were eligible for the present study. Figure 1 also shows the participants who were excluded for different reasons.

### Preterm group

The original study sample included all adolescents born ≤ 32 weeks in the catchment area of Malmö and Lund in southern Sweden from 1994 to 1996 (n = 192). With access to the Swedish Medical Birth Register during this period, information on the children’s gestational age, birth weight, and number of siblings was collected from the Swedish National Board of Health and Welfare.

In this study, the term ‘preterm’ is used to describe children born at 32 weeks or earlier, ‘extremely preterm’ to describe children born at 23 to 28 weeks, and ‘very preterm’ to describe children born at 29 to 32 weeks of gestation [22, 23].

### Full-term control group

In a previous study, a control group of children born full term (born ≥ 37 gestational weeks) was matched with every participating preterm child by age, sex, immigrant background (defined as having at least one parent born outside the Nordic countries), dental clinic and dentist [24]. All of these controls were invited to participate at 12–14 and 17–19 years of age.

### Questionnaire

**Fig. 2:**
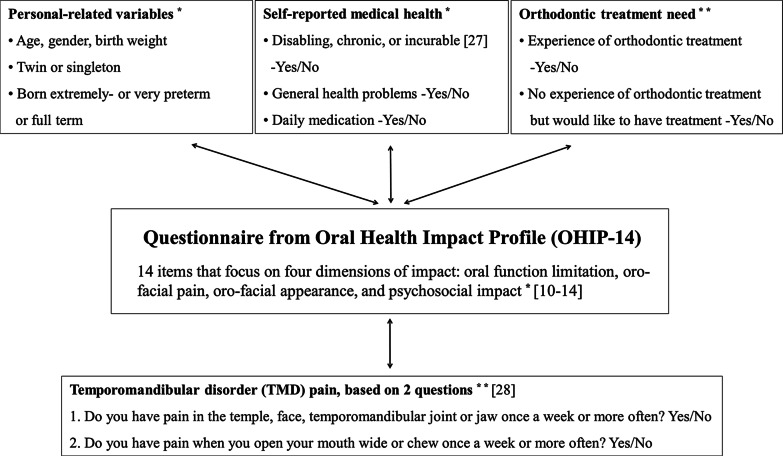
Questionnaire of the relationships between Oral Health Impact Profile (OHIP-14) and independent variables included in the present survey of extremely- or very preterm and full-term adolescents at 12–14 and 17–19 years of age. *Items included in questionnaire at both 12–14 and 17–19 years of age. **Items included in questionnaire at 17–19 years of age

The questionnaire was of a self-report design, including the Oral Health Impact Profile score (OHIP-14) [10–12], to measure OHRQoL and independent variables (Fig. 2). The same questionnaire was used at the age of 12–14 years and 17–19 years [20]. OHIP-14, which is the shorter version of the OHIP-49 [25, 26], comprises 14 items under four domains: oral function limitation, oro-facial pain, oro-facial appearance and psychosocial impact [13, 14].

The participants were asked to respond according to frequency of impact on a 5-point Likert scale coded ‘never’ (score 0), ‘hardly ever’ (score 1), ‘occasionally’ (score 2), ‘fairly often’ (score 3) and ‘very often’ (score 4) using a recall period of one year. Mean scores for each item were calculated and compared within the extremely preterm, very preterm, and full term groups. Adding the response scores for the 14 items gives a total OHIP-14 mean score that ranges from 0 to 56, with higher scores indicating a poorer OHRQoL.

Medical health problems were categorised according to three variables: chronic illness, general health problems and daily medication. Westbom and Kornfält [27] use the following definition for chronic disease: (i) a disorder that is disabling, chronic, or incurable, or (ii) a disorder occurring at least three months during a one-year period and interfering with daily life functioning and/or needing treatment or special aids during at least three months, or (iii) a disorder requiring hospitalisation for at least one month or at least three periods during a one-year period [27]. The definition of general health problems included patients with medical problems of lesser severity or duration, for example, allergies or minor respiratory disorders. For the screening of TMD pain, a validated instrument has been used. This instrument is easy to use with two questions addressing pain in the temporomandibular region. Having TMD pain was defined as answering ‘yes’ to one or both of the two included screening questions shown in Fig. 2 [28].

The participants were asked to respond if they have had experience of orthodontic treatment, and if so, which treatment modality—for example, fixed appliances, removable appliances, or both fixed and removable appliances (yes or no). They were asked to rate their satisfaction with the result of the orthodontic treatment on a 100 mm Visual Analogue Scale. Rating ≥ 70 was classified as good satisfaction and ≤ 30 as less satisfied with orthodontic treatment. Respondents who had not been treated orthodontically were asked if they had a subjective orthodontic treatment need (Fig. 2). If the answer was positive, they were asked to describe in free text the reason for wanting treatment.

### Ethics

The Ethics Committee of the Medical Faculty of Lund University approved the previous studies from which the material was collected (Dnr LU 362–01, Dnr 618/2007) and the present questionnaire study (Dnr Etik H15 2013/39). Written information about the study, including information of full confidentiality and the right to discontinue participation at any time, was posted to the adolescents and their parents, and a written informed consent form was obtained. The questionnaire with a reminder was sent out twice, and a further reminder was conducted by telephone. All methods were performed in accordance with the relevant guidelines and regulations.

### Statistical analysis

As no normative values exist for adolescents on OHIP-14, the analyses were performed in relation to the mean score of the OHIP total. The chi‐square test was used for comparisons between genders and between twins and singletons, within the extremely-, very-, and full-term control groups, respectively. The chi-square test was also used in comparisons between extremely-, very-, and full-term groups regarding three variables of medical health problems: chronic illness, general health problems, and daily medication. The Bonferroni-test was used in comparisons between extremely-, very-, and full-term groups regarding chronic illness to identify where the significant difference was present between the three groups. Further, to contrast the differences of mean scores of OHIP-14 between the groups, the ANOVA test was used for comparisons within and between the study groups of extremely preterm, very preterm and full term-born adolescents**.** Differences at the 5% level of probability were considered statistically significant. The Statistical Package for the Social Sciences (SPSS), version 21.0, 24.0 and 26.0 was used.

## Results

### Questionnaire

A total of 191 adolescents participated in the present survey. Figure 1 shows the flowchart of adolescents in the preterm group (extremely and very preterm-born) and the fullterm control group from the start at the ages of 12–14 and 17–19. The mean age at the time of the questionnaire for those who are between 12–14 years and 17–19 years is shown in Table 1. All participants reported a good OHRQoL measured with OHIP-14 at both 12–14 years and 17–19 years. No significant differences in the comparisons of the total mean scores were revealed between the groups, between gender or in domain-specific scores over the 5-year period (Table 2).

**Table 1:** Baseline characteristics of the study groups of extremely preterm adolescents (EPT), very preterm adolescents (VPT), and full term-born adolescents answering the questionnaire at both 12–14 and 17–19 years of age

	EPT	VPT	Full term
	n = 25	n = 73	n = 93
*Sex*			
Boys	15 (60%)	33 (46%)	43 (46%)
Girls	10 (40%)	40 (54%)	50 (54%)
Mean age (range)	12.6 (11.2–14.0)	12.7 (11.3–14.2)	12.8 (11.2–14.2)
12–14 years of age			
Mean age (range)	18.2 (16.8–19.3)	18.3 (17.0–19.3)	18.4 (16.8–19.9)
17–19 years of age			
Mean gestational age in weeks (range)	26.6	30.8	≥ 37
	(24–28)	(29–32)	No data available
Mean birth weight in grams (range)	893.6	1607.3	3521.0
	615–1250	840–2235	2590–4400
Twins/triplets	2 (8%)	25 (34%)	4 (4%)

**Table 2:** Oral Health Related Quality of Life (OHIP-14) at 12–14 and 17–19 years of age in extremely preterm (EPT), very preterm (VPT), and full term-born adolescents

	EPT	VPT	Full term
	n = 25	n = 73	n = 93
*Oral function limitation* [13, 14]	*Mean (SD)*^*1/*^*Mean (SD)*^*2*^	*Mean (SD)*^*1*^*/Mean (SD)*^*2*^	*Mean (SD)*^*1/*^*Mean (SD)*^*2*^
1. Trouble pronouncing words	0.08 (0.28)/0.28 (0.68)	0.05 (0.23)/0.10 (0.34)	0.11 (0.43)/0.04 (0.20)
2. Decreased sense of taste	0.08 (0.28)/0.04 (0.20)	0.03 (0.16)/0.10 (0.38)	0.03 (0.18)/0.05 (0.27
4. Difficulty chewing	0.60 (0.87)/0.52 (0.92)	0.36 (0.70)/0.38 (0.89)	0.33 (0.68)/0.47 (0.80)
7. Uncomfortable when eating	0.12 (0.33)/0.08 (0.28)	0.14 (0.51)/0.15 (0.46)	0.05 (0.23)/0.16 (0.47)
8. Avoids eating	0.12 (0.33)/0.08 (0.28)	0.18 (0.51)/0.18 (0.61)	0.15 (0.42)/0.19 (0.52)
*Oro-facial pain* [13, 14]			
3. Painful aching	0.28 (0.54)/0.28 (0.54)	0.53 (0.71)/0.68 (0.88)	0.38 (0.64)/0.62 (0.77)
*Oro-facial appearance* [13, 14]			
5. Self-conscious	0.16 (0.47)/0.56 (0.87)	0.25 (0.62)/0.38 (0.89)	0.23 (0.55)/0.32 (0.6
10. Been embarrassed	0.12 (0.33)/0.36 (0.99)	0.25 (0.60)/0.38 (0.84)	0.29 (0.60)/0.37 (0.70)
*Psychosocial impact* [13, 14]			
6. Felt tense	0.20 (0.82)/0.28 (0.61)	0.16 (0.47)/0.23 (0.59)	0.13 (0.42)/0.22 (0.60)
9. Difficulty in relaxing	0.20 (0.41)/0.12 (0.44)	0.11 (0.36)/0.23 (0.61)	0.11 (0.35)/0.28 (0.63
11. Irritable with others	0.00 (0.00)/0.08 (0.28)	0.11 (0.46)/0.03 (0.23)	0.11 (0.38)/0.10 (0.36)
12. Difficulty doing jobs	0.08 (0.40)/0.04 (0.20)	0.03 (0.23)/0.07 (0.30)	0.04 (0.20)/0.08 (0.30)
13. Found life unsatisfying	0.08 (0.40)/0.08 (0.28)	0.12 (0.58)/0.16 (0.65)	0.06 (0.29)/0.17 (0.58)
14. Unable to function in daily life	0.00 (0.00)/0.00 (0.00)	0.00 (0.00)/0.08 (0.49)	0.01 (0.10)/0.02 (0.15)
*Total scores*			
Mean	2.12 (2.82)/2.80 (3.70)	2.32 (3.73)/3.16 (5.13)	2.03 (2.96)/3.10 (4.88)
Median	1.00/2.00	1.00/1.00	1.00/1.00
Range	0–11/0–14	0–20/0–30	0–16/0–32

Mean (SD)^1^ = at 12–14 years of age. Mean (SD)^2^ = at 17–19 years of age. No significant differences were found within and between the groups over the five year period (ANOVA test)

In comparisons within the groups of extremely preterm, very preterm, and full-term adolescents at 12–14 years of age, girls reported higher total mean scores of OHIP-14 in all groups; extremely preterm 2.30 (SD 3.43), very preterm 2.78 (SD 4.55) and full-term adolescents 2.08 (SD 2.86) in comparison with boys; extremely preterm 2.00 (SD 2.45), very preterm 1.76 (SD 2.32) and full-term adolescents 1.98 (SD 3.01), however, there was a non-significant difference compared with boys. In comparisons within the groups at 17–19 years of age, corresponding figures for girls were; extremely preterm 4.70 (SD 4.83), very preterm 3.55 (SD 6.12) and full-term adolescents 3.48 (SD 5.35), a non-significant difference compared with boys; extremely preterm 1.53 (SD 2.03), very preterm 2.70 (SD 3.61) and full-term adolescents 2.65 (SD 4.29).

Extremely preterm-born twin/triplets reported higher total mean scores of OHIP-14 than singletons at 12–14 years of age (3.50 (SD 0.71) vs 2.00 (SD 2.91); however, this is a non-significant difference.

### Medical health problems

Chronic illness increased during the 5-year period in all groups. Further, it was more frequently reported among the extremely- and very preterm-born adolescents than in the full-term control group, in total a statistically significant difference at both 12–14 (*p* = *0.027***)** and 17–19 years of age (*p* = *0.017***)** (Table 3). However, comparisons between extremely and very preterm group (*p* = *0.351*), extremely preterm with full-term group (*p* = *0.060*), and very preterm with full-term group (*p* = 1.000), revealed no statistically significant differences regarding chronic illness at 12–14 years of age. At 17–19 years of age, similar comparisons between the three groups showed statistically significant difference between extremely preterm and full-term group (*p* = *0.033)*. Further, a non-significant difference between extremely preterm and very preterm group (*p* = *0.063*), and very preterm with full-term group (*p* = *1.000*) (Table 3). The very preterm group with chronic illness reported significantly higher total mean scores of OHIP-14, compared to those adolescents without chronic illness at 12–14 years of age (*p* = *0.015*) (Table 4).

**Table 3:** Medical health problems (chronic illness, general health problems and daily medication) in extremely preterm (EPT), very preterm (VPT), and full term-born adolescents at 12–14 and 17–19 years of age. Chi-square test. Further, participants with experience of orthodontic treatment and claimed orthodontic treatment need in extremely preterm (EPT), very preterm (VPT) and full term-born adolescents at 17–19 years of age

	12–14 years of age				17–19 years of age			
	EPT	VPT	Full term	*p* value	EPT	VPT	Full term	*p* value
	N = 25	N = 73	N = 93		N = 25	N = 73	N = 93	
Chronic illness	5 (20%)	5 (7%)	4 (4%)	0.027*	9 (36%)	10 (14%)	12 (13%)	0.017**
General health problems	7 (28%)	20 (27%)	18 (19%)	*0.426*	7 (28%)	21 (29%)	20 (22%)	*0.571*
Daily medication	4 (16%)	7 (10%)	7 (8%)	*0.418*	6 (24%)	13 (18%)	10 (11%)	*0.192*
*Experience of orthodontic treatment*					9 (36%)	28 (38%)	26 (28%)	
Fixed appliances					6 (67%)	15 (56%)	15 (58%)	
Removable appliances					1 (11%)	5 (19%)	7 (27%)	
Both fixed and removable appliances					2 (22%)	7 (26%)	2 (8%)	
Missing value						1 (1.5%)	2 (8%)	
Good satisfaction with orthodontic treatment (rating ≥ 70 on the VAS scale)					7 (78%)	21 (75%)	22 (85%)	
No experience of orthodontic treatment, but the participant wants to have treatment					4 (16%)	7 (10%)	18 (19%)	
*Marked comments of why there is need for orthodontic treatment*								
Rotated teeth					0 (0%)	4 (57%)	7 (39%)	
Spacing					0 (0%)	1 (14%)	5 (28%)	
Crowded teeth					1 (25%)	0 (0%)	2 (11%)	
To optimise tooth-brushing					0 (0%)	0 (0%)	1 (6%)	
Several reasons, e.g., overjet, crowded teeth, specific esthetic reasons					3 (75%)	2 (29%)	1 (6%)	
Missing value							2 (11%)	

*Comparison EPT with VPT *p* = 0.351, EPT with Full term *p* = 0.060 and VPT with Full term *p* = 1.000—Bonferroni-test

**Comparison EPT with VPT *p* = 0.063, EPT with Full term *p* = 0.033 and VPT with Full term *p* = 1.000—Bonferroni-test

**Table 4:** Comparison of oral health-related quality of life (OHIP-14) within the three study groups (extremely preterm (EPT), very preterm (VPT) and full term-born adolescents). The groups compared are individuals with and without chronic illness at 12–14 and 17–19 of age, and TDM pain or not according to two screening questions at 17–19 years of age. Further, comparisons regarding subjective orthodontic treatment need or not at 17–19 years of age. ANOVA test

	OHIP-14 – EPT	OHIP-14 – VPT	OHIP-14 – Full term
	N = 25	N = 73	N = 93
	Mean score ± SD	Mean score ± SD	Mean score ± SD
Chronic illness 12–14 years	Yes (N = 5) 3.20 ± 3.11	Yes (N = 5) 6.20 ± 5.22	Yes (N = 4) 0.50 ± 1.00
	No (N = 20) 1.85 ± 2.76	No (N = 68) 2.03 ± 3.48	No (N = 89) 2.10 ± 3.00
*p* value	*0.349*	***0.015***	*0.292*
Chronic illness 17–19 years	Yes (N = 9) 2.56 ± 4.77	Yes (N = 10) 5.80 ± 8.88	Yes (N = 12) 1.58 ± 1.44
	No (N = 16) 2.94 ± 3.11	No (N = 63) 2.75 ± 4.22	No (N = 81) 3.32 ± 5.17
*p* value	*0.810*	*0.080*	***0.017***
TMD pain^1^ 17–19 years	Yes (N = 6) 4.33 ± 5.72	Yes (N = 9) 9.89 ± 10.11	Yes (N = 21) 4.86 ± 8.06
	No (N = 19) 2.32 ± 2.85	No (N = 64) 2.22 ± 3.11	No (N = 72) 2.58 ± 3.37
*p* value	*0.438*	*0.053*	*0.221*
TMD pain^2^ 17–19 years	Yes (N = 2) 0.00 ± 0.00	Yes (N = 6) 15.00 ± 9.859	Yes (N = 10) 9.40 ± 10.44
	No (N = 23) 3.04 ± 3.76	No (N = 67) 2.10 ± 2.73	No (N = 83) 2.34 ± 3.06
*p* value	*0.273*	***0.024***	*0.062*
Subjective orthodontic treatment need 17–19 years	Yes (N = 4) 6.75 ± 5.50	Yes (N = 7) 6.14 ± 10.75	Yes (N = 18) 4.06 ± 4.45
	No (N = 12) 0.92 ± 1.73	No (N = 38) 1.74 ± 3.06	No (N = 48) 2.27 ± 4.05
*p value*	***0.011***	***0.031***	*0.215*

TMD pain1 – Do you have pain in the temple, face, temporomandibular joint or jaw once a week or more? TMD pain2 – Do you have pain when you open your mouth wide or chew once a week or more often

### TMD pain

The two screening questions to identify TMD pain registered significantly higher mean scores on OHIP total (Table 4). Those who experience TMD pain once a week or more when opening wide or chewing often showed a significantly higher mean score on the OHIP total in the very preterm group compared to those adolescents without TMD pain (*p* = *0.024*) (Table 4).

### Orthodontic treatment need

Of all the participants, 63 (33%) have had orthodontic treatment at the age of 17–19 years. Of those, 9 (36%) were extremely preterm-born, 28 (38%) were very preterm-born, and 26 (28%) were full term-born (Table 3). Of all the participants 29 (15%) had a subjective orthodontic treatment need at 17–19 years and the participants main reasons for wanted treatment are listed in Table 3.

Regarding OHRQoL, the results show that significantly worse total OHIP-14 mean scores were revealed in the groups of extremely (*p* = *0.011*) and very preterm-adolescents (*p* = *0.031*) with subjective orthodontic treatment need compared to those adolescents without orthodontic treatment need at 17–19 years of age (Table 4).

## Discussion

The present study is the first to evaluate the OHRQoL, measured with OHIP-14, of extremely- and very preterm-born and full term-born adolescents. This approach is aimed at exploring oral health perspectives related to prematurity, as compromised health and functioning are often reported in cases of prematurity. This 5-year longitudinal study did not reveal any overall differences in mean scores of OHRQoL between the groups, which is a sustainable result. However, the poor OHRQoL measured with OHIP-14 in very preterm adolescents at age 12–14 was found to be due to chronic illness and at age 17–19 to TMD pain. Additionally, extremely- and very preterm-born adolescents with a subjective orthodontic treatment need also reported a poor OHRQoL.

Regarding the hypotheses of the study, the OHRQoL was predicted to be impaired over the 5-year period, but this was not found, thus disproving the proposed hypothesis that this would be the case due to persistent medical health problems (chronic illness, general health problems and daily medication). Chronic illness increased in all groups during the 5-year period, and significantly more illnesses were reported among the extremely- and very preterm-born adolescents than in the control group during both the ages of 12–14 years and 17–19 years. This is in agreement with the literature reporting an association of preterm birth with difficulties in areas such as motor skills, learning, behaviour in preschool to beyond school age years [2], an increased risk of, for example, pulmonary arterial hypertension later in life [29], and persistent airway obstructions in those born extremely preterm from mid-childhood to adulthood [30]. The central question of the issue of this study was, did the burden of illness and difficulties during adolescence affect OHRQoL? For the very preterm-born group, this was evident, as there were significantly higher mean scores of OHIP-14 at 12–14 years of age compared to those without chronic illness. Gender had no effect on OHIP-14 scores, which is in line with other studies with participants of similar ages [31].

The questions on pain have been validated in adolescents with very good reliability and high validity in adolescents aged 12–19 years [28]. The very preterm group with TMD pain (‘yes’ to one or both screening questions) had significantly higher mean scores of OHIP-14 than those without TMD pain, thus confirming the proposed hypotheses. In fact, the highest mean scores of OHIP-14 in this study were revealed regarding TMD pain. Another study with the same participants showed that 23% of the extremely- and very preterm adolescents have TMD pain, and within these groups, many reported trouble sleeping, stomach pain, and feelings of hopelessness about the future [32]. TMD pain has been reported to be associated to OHRQoL in a multidimensional approach, and the presence of physical and psychological variables, social function and level of well-being may also increase the development of impaired OHRQoL [33].

Subjective orthodontic treatment need and impaired OHRQoL is frequently studied [18, 34]. However, to our knowledge, no studies exist that focus on very- and extremely preterm-born individuals. Therefore, there is a growing interest in the relationship between malocclusion, subjective treatment need and OHRQoL [34]. Measuring the OHRQoL in this context has become increasingly essential because the patient’s perception and opinion is of vital importance for overall satisfaction and to improve the dentist–patient relationship, thus achieving more successful treatment results. Most of the participants in this survey reported a good level of satisfaction; however, the mean scores revealed a need for orthodontic treatment for those who have not had treatment.

The instrument of the Oral Health Impact Profile (OHIP) is used extensively for the assessment of OHQoL. OHIP is conducted in different versions (OHIP-14, 5 and 49), with the same dimensions across all three scales. The four dimensions Oral Function, Oro-facial Pain, Oro-facial Appearance and Psychosocial Impact cover OHRQoL, and are relatively newly established [13, 14]. We found the four dimensions relevant and useful, and to our knowledge this study is one of the first using this four-dimension assessment in preterm born individuals. As there is no normative value for adolescents on OHIP-14, analyses and comparisons were made in relation to the mean score; however, reference values in adolescents are needed for interpretation. The mean scores do not give any detailed information of the OHQoL, and a reference value is difficult to find. However, the single questions elucidate what kind of problems exists. In this study, the highest mean score of OHIP-14 was revealed in the extremely preterm group regarding difficulty chewing, in the very preterm group for painful aching, and equal in the control group for difficulty chewing and painful aching. However, no mean scores indicated problems affecting daily life or any significant differences between the groups. In general, the OHIP-14 measures the influence of oral disease on the individual’s social function [35]; however, no positive dimensions rather issues of discomfort, dysfunction, and disability are measured in attempt to capture all possible functional and psycho-social outcomes of oral disorders. For adolescents, this must be explored because this age involves a transition to adulthood with expectations of being able to manage their own lives in different ways, including taking responsibility for their oral health. For the dental professionals, the potential use of subjective health status measures is important for predicting treatment need and essential for the allocation of health resources.

Little is known about OHRQoL in preterm-born individuals; however, to the best of our knowledge, this survey is the first of its kind, with aspects worthy of study given that the very- and extremely preterm group has a compromised health status with lifelong consequences for health, growth and development. The study is of a longitudinal design, including all extremely- and very preterm-born adolescents born at the university hospitals of Lund and Malmö during a 3-year period. All the adolescents had participated in several previous studies [20–24, 32], and repeated requests for participation might be tiring for the participants and the families; therefore, the number of participating adolescents must be appropriate. Further, the assessments were made by the same researcher (SBR) on both occasions (12–14 years and 17–19 years of age).

Another strength in the assessment of OHRQoL in this study is the choice of OHIP-14 as an instrument, as it is widely used, which offers international comparability. It is also well structured and tested and has shown to have good reliability and validity in the general population [36]. It was originally developed for adults but has since been applied to adolescents because, during the adolescent years, the individual has the capacity to think abstractly and is able to relate good or bad experiences when recalling the past. No normative values are known for adolescents or for extremely- and very preterm-born individuals, but from a professional ethics approach, these values are necessary in order to indivudalise and explore each individual viewpoint and condition with certainty.

A limitation of the study is the small number of the extremely preterm-born adolescents (n = 25) who participated, which made comparisons with the very preterm and full-term group difficult. However, the number of those extremely preterm-born is in line with official statistics in Sweden, even though it is a limited number compared to the very preterm group. To increase that number, it would have been necessary to include a larger total sample of preterm-born individuals. As this is a follow-up study, it was not possible to redesign the study with this particular point of view. To generate a clearer picture of all the participants’ OHRQoL, additional information about their oral/dental health status, for example, their caries prevalence, could be used to analyse the impact of different clinical variables on OHRQoL. This information was collected from all the participants’ dental records used in an ongoing study at Malmö University.

Another limitation of the current study is the lack of information regarding the participants’ physical condition, such as weight, height and muscle mass index. This must be considered valuable information in the perspective of describing how malocclusions impact on general health and oral health-related quality of life. However, given that a questionnaire is not the ideal way to collect information regarding weight, height and muscle mass index, this was not included in the questionnaires due to the unreliability of the information, which is a shortcoming.

The aim of this study was to provide knowledge about and to study the effects of OHRQoL in extremely and very preterm born adolescents over a 5-year period. To assess a wider dimension of OHRQoL, additional studies are required, for example, those of a qualitative study design with an analysis of how prematurity may affect OHRQoL across the life course, especially in the extreme preterm group. The result would provide a valuable contribution of understanding and predicting future dimensions of life satisfaction in these extremely preterm survivors. This type of study is appropriate for this specific group due to the group’s increased risk for health problems and developmental vulnerability throughout childhood to young adulthood.

## Conclusions

The present study showed that all adolescents reported an overall good self-perceived OHRQoL. Furthermore, poor OHRQoL measured with OHIP-14 in very preterm adolescents was related to chronic illness aged 12–14, and aged 17–19 to TMD pain. It was also related to subjective orthodontic treatment need in extremely- and very preterm-adolescents at 17–19 years of age. These results indicate the value of further evaluating OHRQoL in larger cohorts of adolescents and young adults born extremely and very preterm.

## Data Availability

The datasets used and/or analysed during the current study are available from the corresponding author on reasonable request.

## Abbreviations

OHRQoL: Oral health-related quality of life
OHIP-14: Oral health impact profile
TMD: Temporomandibular disorder
EPT: Extremely preterm (born in gestational weeks 23–28)
VPT: Very preterm (born in gestational weeks 29–32)
SD: Standard deviation
